# The Subjective Visual Vertical and the Subjective Haptic Vertical Access Different Gravity Estimates

**DOI:** 10.1371/journal.pone.0145528

**Published:** 2015-12-30

**Authors:** Lindsey E. Fraser, Bobbak Makooie, Laurence R. Harris

**Affiliations:** Center for Vision Research, York University, Toronto, Ontario, Canada; University of Amsterdam, NETHERLANDS

## Abstract

The subjective visual vertical (SVV) and the subjective haptic vertical (SHV) both claim to probe the underlying perception of gravity. However, when the body is roll tilted these two measures evoke different patterns of errors with SVV generally becoming biased towards the body (A-effect, named for its discoverer, Hermann Rudolph Aubert) and SHV remaining accurate or becoming biased away from the body (E-effect, short for Entgegengesetzt-effect, meaning “opposite”, i.e., opposite to the A-effect). We compared the two methods in a series of five experiments and provide evidence that the two measures access two different but related estimates of gravitational vertical. Experiment 1 compared SVV and SHV across three levels of whole-body tilt and found that SVV showed an A-effect at larger tilts while SHV was accurate. Experiment 2 found that tilting either the head or the trunk independently produced an A-effect in SVV while SHV remained accurate when the head was tilted on an upright body but showed an A-effect when the body was tilted below an upright head. Experiment 3 repeated these head/body configurations in the presence of vestibular noise induced by using disruptive galvanic vestibular stimulation (dGVS). dGVS abolished both SVV and SHV A-effects while evoking a massive E-effect in the SHV head tilt condition. Experiments 4 and 5 show that SVV and SHV do not combine in an optimally statistical fashion, but when vibration is applied to the dorsal neck muscles, integration becomes optimal. Overall our results suggest that SVV and SHV access distinct underlying gravity percepts based primarily on head and body position information respectively, consistent with a model proposed by Clemens and colleagues.

## Introduction

The perceived direction of gravity is important because of the pervasive use of gravity as a reference for almost all aspects of perception and action. Perceived orientation is fundamental for recognizing and categorizing objects as well as for balance and orienting oneself in the environment [1]. However, measuring the perceived direction of gravity and interpreting such measurements is complex because the measured direction depends heavily on the methodology employed. Two common methods that have been used extensively to assess the perceived direction of gravity are the subjective visual vertical (SVV) and the subjective haptic vertical (SHV). In these tasks, participants are asked to judge the position of a visual or haptic stimulus relative to gravity. Curiously, when a participant is tilted, these two measures give different estimates (see below). How are such differences to be understood? Schuler et al. [2] found that trial-to-trial variability of SVV and SHV was correlated across different magnitudes of roll tilt from which they argued that both measures access a common underlying estimate of the gravity vector, with modality-specific influences responsible for their different biases. In contrast, Clemens et al. [3] modeled gravity perception as having two distinct egocentric reference frames, one inferring the position of the gravity vector using sensory information about the head’s position in space, the other from sensory information about the body in space. We performed a series of five experiments to determine the appropriate framework in which gravity perception is represented in SVV and SHV tasks.

The SVV has been taken as a definitive estimate of the perceived direction of gravity since its introduction by Aubert [4]. When standing upright, participants are able to set the orientation of a luminous line to within ± 2° of gravitational vertical [5,6], but when the body is rolled there is a small bias away from gravitational vertical either in the same direction as the tilt [4,7–11] or in the opposite direction [3,12–14]. The direction depends partly on the amount of tilt and partly on the details of the methodology. A tendency to revert to a body-defined upright (on the assumption, or “prior”, that the body is always upright) may contribute to a bias towards the body [15,16], whereas a failure to completely compensate for ocular counter-roll (OCR) may contribute to biases away from the body ([17,18]; but see [19]).

The SHV was introduced to overcome possible errors in the SVV due to uncompensated OCR and to access the “true” internal representation of the direction of gravity. The SHV shows a pattern of errors that is rather different from the SVV, with a bias away from the body’s tilt ([2,20]; but see [21]) and a vulnerability to hysteresis (where different initial positions of the hand change the response).[22]. Whether this or the SVV or some combination represents a unique “true” representation remains to be determined.

**Fig 1:**
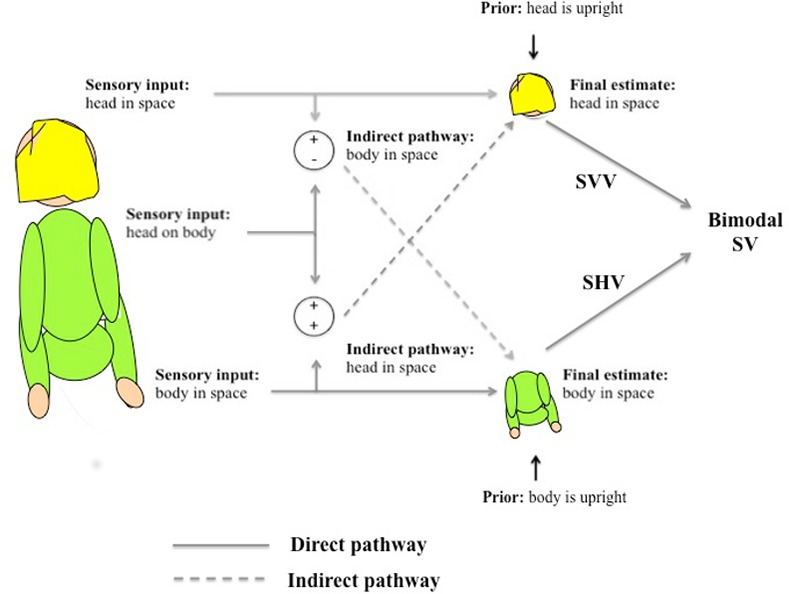
A simplified version of the model presented in Clemens et al [3], with several additions. The original model posits two final position estimates used to determine the direction of gravity, one based on the head’s position (with indirect input from body-based sensors, converted via neck proprioception into head-centric coordinates) and the other on the body (with indirect input from the head). The authors suggest SVV uses the head-based gravity estimate; here we argue that SHV accesses the body-based estimate.

When only the head is tilted (with the body upright) SHV is accurate but biases in SVV are still found [11] which suggests that head tilt may drive SVV errors, but not SHV errors. SHV errors, in contrast, may be driven by the tilted position of the torso and limbs; this is supported by Bauermeister et al. [20] who found that SHV shifts position depending on which hand is used to feel the test probe. Schuler and colleagues [2] have suggested that different errors in SVV and SHV may reflect modality-specific errors, such as the hand’s position being misestimated if the tilt and torsion of the joints of the arm are misperceived. According to this theory, errors in SVV and SHV may have more to do with errors in comparing the test probe to an internal estimate of gravity than to errors in the actual estimate itself. In other words, response errors could reflect misperception of the *probe’s* orientation as sensed by a specific modality, not the orientation of gravity. Such modality specific errors are ostensibly head based for vision and limb/torso based for haptic measures. An alternative theory by Clemens and colleagues [3] suggests that there may be multiple, simultaneous internal estimates of gravity, held in different reference frames (head based or body based) that may not necessarily be congruent and which may be differentially accessed by different verticality judgment tasks (see Fig 1). If this is the case, and if SVV and SHV access different estimates within this system, then SVV and SHV should be able to combine in a statistically optimal fashion into a bimodal estimate of gravity. That is, if the noise in SVV and SHV estimates are uncorrelated then a bimodal estimate of gravitational vertical should be the average of visual and haptic responses, weighted by the respective reliabilities of the two measures, with an increased precision compared to unimodal responses [23,24]. In experiments 4 and 5 we test whether a bimodal probe of subjective vertical is consistent with these predictions of optimal cue combination.

The aim of this study is to test whether SVV and SHV access different underlying gravity estimates, similar to the head- and body-based estimates described by Clemens et al. [3], as shown diagrammatically in Fig 1. We test this by directly comparing SVV and SHV in the same participants using a robust response methodology (experiment 1). Previous studies suggest that the two measures produce different apparent verticals (reviewed above); replicating this difference in the same subjects would suggest separate mechanisms for haptic and visual estimates of gravity. To probe these mechanisms further, we test whether head and trunk orientation have differential influences on SVV and SHV (experiment 2), and whether disrupting of a head-based vestibular signal differentially affects the two measures (experiment 3). A stronger influence of head-based and vestibular cues on SVV and a stronger influence of the body on SHV would be compatible with the notion that the SVV is based on a head-based estimate of gravity, while the SHV is based on a body-based estimate. Finally, if separate underlying estimates of gravity for SVV and SHV exist, integration of the SVV and SHV into a single, bimodal task should be statistically optimal according to a Maximum Likelihood Estimate (MLE) model of cue integration (experiments 4 and 5). In providing support for the two-estimate model, we also hope to shed light on the seemingly modality-specific biases previously reported in the literature.

## General Methods

### Participants

All participants were members of the York community. Participating undergraduate students received a bonus credit towards their *Introduction to Psychology* course, other participants did not receive compensation. All participants had normal or corrected-to-normal visual acuity and reported no history of dizziness or balance disorders. The details of participants in each experiment (number of participants, age range, sex, handedness) are reported in the experiment-specific methods sections below. All the experiments were approved by the ethics board of York University and run according to the principles of the Declaration of Helsinki (the Declaration of Helsinki was originally adopted in 1964 and has been revised multiple times since then, the last being 2013. It is a living document, but the basic type of non-invasive research we are doing here is covered by the original version see http://www.wma.net/en/30publications/10policies/b3/). Written consent was obtained prior to participation (a procedure approved by the ethics board at York University).

### Apparatus

#### Test rod

A hollow glass rod (30.5 cm long, 0.9 cm dia) was fitted with a blue cold-cathode light so that it could be illuminated from the interior. The rod was mounted orthogonal to the shaft of a motor (Applied Motion Products 23Q-3aE Integrated Stepper Motor) that had a precision of 20,000 steps per revolution. When illuminated the rod formed the stimulus for the SVV but it could also be touched to provide the stimulus for the SHV. The motor emitted minimal noise while changing position that did not reflect the direction of movement. The motor was covered by a circular sheet of black card (diameter 61 cm) to avoid it being illuminated by the light from the rod and thus provide unwanted visual orientation cues. The rod was viewed through a circular shroud (diameter 12.7 cm) that occluded peripheral vision. The entire apparatus was adjusted so that the center of the rod was aligned with the nose of each participant with a viewing distance of 40 cm. The apparatus was arranged to provide the participant free access to the rod with their right hand.

#### Platform

**Fig 2:**
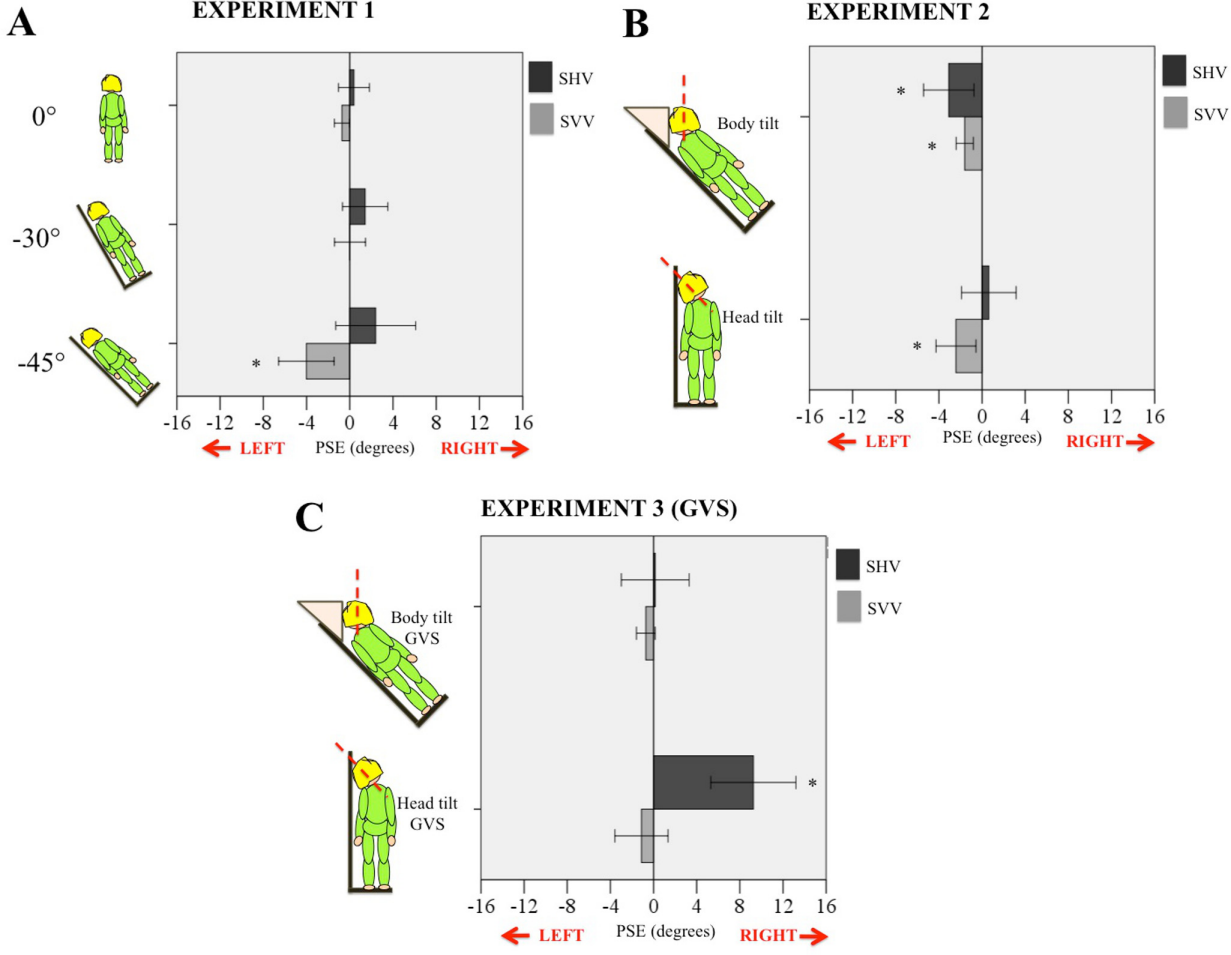
Results for experiments 1–3. A. Experiment 1. Mean PSEs for SHV (black) and SVV (grey) for each of three body tilts (0°, 30° and 45°, shown as insets on the left of histogram) where 0° corresponds to gravitational vertical. B. Experiment 2. Mean PSEs for SHV and SVV for head and body tilt (see insets on the left). C. Experiment 3. Mean PSEs for SHV and SVV for head and body tilt with disruptive GVS. Asterisks indicate mean was significantly different from 0° at p<0.05. Error bars are 95% confidence intervals, which make clear which conditions included an accurate perception of gravitational vertical.

Participants stood or lay against a wooden platform set at one of three angles (0°, 30° left or 45° left), so that their body midline was upright or tilted in the roll plane. A second board was attached perpendicularly at the base to support participants’ feet and a stiff, square pillow was used to support the head such that it lay parallel to the board surface (as measured visually by the experimenter). Additional pillows for the neck and hips were used to ensure that participants could comfortably keep their head still in this position without being strapped down. In the 0° condition participants were instructed to keep their head upright during the task (see insets to Fig 2).

### Procedure

#### SVV

On each trial the motor drove the rod to a test orientation selected by adaptive staircase algorithm, which chooses orientations based on previous responses in order to quickly hone in on the point of subjective equivalence (PSE). We used the QUEST procedure for experiments 1–3, and the psi-method for experiments 4–5 (see details below). The rod was illuminated 1s after reaching the test position. A 400 Hz beep notified the participant it was time to respond. To reduce the risk of potential response artifacts arising from the use of an adjustment technique [25] we used a forced-choice paradigm in which participants responded as to whether the probe was perceived as tilted “left” or “right” of gravitational vertical (i.e., “Would the rod tip over to the left or to the right?”). They indicated their response with their right hand using the buttons on a mouse held in their left hand. The light was then turned off and the rod moved to the next test orientation. There were no time restrictions on any trials; participants took roughly 1–3s to respond on each SVV trial.

#### SHV

Participants were instructed to keep their eyes closed throughout the task. The motor drove the rod to the chosen test orientation, but the rod did not illuminate. A 400 Hz beep sounded to prompt the participant to reach out with the right hand and feel the orientation of the rod using a pincer grip (participants were allowed to move their hand along the rod as they liked, provided they kept this hand configuration). They then indicated their response (“left” or “right”) with the right hand by pressing the left or right buttons on a mouse held in their left hand. This required them to stop feeling the rod to respond. The rod was then moved to the next test orientation and the next trial began. There were no time restrictions on SHV trials; participants took roughly 3–5 s to respond on each trial.

0° is defined as true gravitational upright. Positive values indicate a clockwise tilt and negative values indicate counterclockwise tilt from the point of view of the participant. In our convention therefore participants were tilted at 0°, -30° or -45° with their left ear down.

## Experiment 1: Comparison of SVV and SHV during Whole-Body Tilt

In experiment 1 we directly compare SVV and SHV at three levels of roll tilt (0°, -30° and -45°) using a within-subjects experimental design and an adaptive staircase algorithm (QUEST, [26]).

### Methods

#### Participants

Forty-eight participants (aged 18–27, 28 female) took part in this experiment. Three self-identified as left-handed. A plot of individual scores showed the PSEs of the left-handers fell within the same range as those of right-handers and therefore both left- and right-hander scores were included in the analysis (see also [22,27]).

#### Procedure

Participants completed SVV and SHV measurements at one of three tilts: 0° (upright), 30° left or 45° left. Experiments were performed in a block design with block order randomized across participants. Test orientations were chosen by a QUEST adaptive staircase [26]. The QUEST was given initial estimates of 0° (no bias) and an initial standard deviation of ±20°. The grain (step size) was set to 0.1°. The QUEST program calculated participants’ PSE estimates based on a psychometric curve constructed from all a participant’s previous responses. The staircase ran for 60 trials in each block (SVV or SHV). In addition, there were 10 SVV training trials run at the beginning of the experiment to familiarize participants with the test paradigm and ensure they understood the instructions. Breaks were offered between blocks. The experiment took approximately 50 minutes to complete.

### Results

Mean SVVs and SHVs for the tested tilts are plotted in Fig 2A. A mixed-model ANOVA of PSEs found a modality x tilt interaction was significant, *F*(2,45) = 4.72, *p* = 0.014. There was also a main effect of modality (visual or haptic), *F*(1,45) = 14.07, *p* = 0.001. Thus, the type of measure used (SVV or SHV) differentially shifted perception depending on the amount of whole-body tilt. Fig 2A shows that the mean SVV tended to tilt further towards the body as tilt increased while SHV tended to tilt further away from the body. Of the six conditions, only the SVV at -45° was significantly different from gravity vertical, *t*(15) = -3.34, *p* = 0.03. There was no significant correlation between the SVV and SHV across 0°, -30° and -45° conditions (p = 0.73).

### Discussion

Experiment 1 directly compared the SVV and SHV with the aim to replicate different patterns of biases in the two measures within the same individuals using a robust psychophysical testing paradigm. Our results show that, within the same individuals, visual and haptic subjective verticals differ and these measures are not correlated across levels of tilt. Similar to previous studies, the SVV was biased towards the body [4,7–11] and the SHV estimates trended in the opposite direction [3,12–14]. Importantly, the ANOVA showed a significant modality x tilt interaction, suggesting that the SVV showed errors increasingly distinct from SHV as tilt increased.

Although we only tested tilts to the left, previous studies suggest that rightward tilt would produce symmetrical data [11,20] and our aim here was only to determine whether SVV and SHV biases differed or were correlated within subjects. The haptic stimulus was always felt with the right hand, which Bauermeister and colleagues [20] have shown may lead to a consistent slight leftward shift–here corresponding to a bias towards the body and hence potentially reducing the observed SHV bias away from the body, a bias that has been reported in other studies [20,2].

Although clearly different, the precise nature of the differences between modalities and their respective subjective verticals remains unclear. Schuler and colleagues [2] found trial-to-trial variability of SHV and SVV were correlated over a range of roll tilts, suggesting that SHV and SVV may share an underlying mechanism for the estimation of gravity. Why, then, do the biases produced by each measure differ? Is it due to modality-specific errors, or to separate gravity estimates, as suggested by Clemens et al., [3]? In experiment 2, we look at the relative influence of the position of the head and body on SVV and SHV to determine whether different errors stem from shared or disparate origins.

## Experiment 2: Comparing SVV and SHV when the Head and Body Are Tilted Separately

Previous studies suggested that SVV errors might be more closely linked to tilts of the head [11]. In contrast, SHV may depend more on the body as head tilt does not evoke SHV errors [28]. Here we independently varied the tilt of the body and head with respect to gravity in the same subjects and measured SVV and SHV.

### Methods

#### Participants

Sixteen participants (aged 18–61, 9 female) took part in this experiment. All participants were naïve to the experiment and did not participate in experiment 1. All self-identified as right handed.

#### Platform

Two body positions were used in this experiment; body tilted and head upright (“body tilt”) or body upright and head tilted (“head tilt”). In the body tilt position, participants lay on their left side supported by the platform described above at a tilt of 45° with a stiff triangular pillow propping the head so that it was aligned with gravity (0°). In the head tilt position, participants stood against the upright platform with their head tilted to the left by 45° (see inset of Fig 2B). Head angles were measured with a head-mounted protractor and spirit level, which were referenced to a plumb line. Other details are as for experiment 1.

#### Procedure

Participants completed SVV and SHV measurements in both positions, yielding four test blocks which were randomly ordered but, due to the difficulty of moving between the tilted and upright platforms, the two body tilt tasks were completed together and the same for the two head tilt tasks, with a break in between. For each block, the QUEST (with the same initial test parameters as for experiment 1) was configured to run for 40 trials. Staircase assessment and subsequent simulations following experiment 1 showed the final 20 trials of the QUEST did not differ substantially in their PSE estimates; thus in the interest of time the number of trials was reduced from 60 to 40 for all participants in this experiment. The experiment consisted of 160 trials, which took approximately 40 minutes to complete.

### Results

Mean SVVs and SHVs for each test condition are plotted in Fig 2B with 95% confidence intervals. A 2x2 repeated measures ANOVA found a significant interaction of modality x position, *F*(1,15) = 5.80, *p* = 0.029, but no main effect of modality or position. T-tests were used to compare PSE scores in each condition to true gravitational vertical (i.e., 0°). In the body tilt condition, both SVV and SHV were significantly tilted to the left *t* (15) = -4.31, *p* = 0.004 (SVV) and *t* (15) = -2.81, *p* = 0.019 (SHV); in the head tilt position, only SVV showed a significant tilt, *t* (15) = -2.79, *p* = 0.019. That is, while tilting the head or body left both evoked a leftward tilt in SVV but only body tilt produced a bias in SHV. Curiously, this SHV bias was in the opposite direction to the trend in SHV found in experiment 1 (Fig 2A) during whole body tilt. We performed two correlation analyses between gravity estimates of SVV and SHV, one for the head tilt, and one for the body tilt. Neither correlation was significant (p = 0.08 and p = 0.59, respectively).

### Discussion

The goal of this experiment was to identify whether the SVV and SHV were differentially affected by tilts of just the head or just the body. Previously, Guerraz et al. [29] showed that both head and body tilt drive SVV errors. Tarnutzer and colleagues [30] further showed that SVV is likely coded in a head-fixed reference frame, and that SVV is most precise and accurate when the head is upright. In our experiment, the SVV was significantly shifted to the left when either the body or the head were independently tilted, suggesting contributions of perceived relative body position to SVV, as well as the head. This does not contradict the claim that SVV is coded in a head-fixed reference frame, but does suggest that perceived trunk orientation is factored into this coding.

In contrast to SVV, the SHV was only biased by a tilt of the body and was unaffected by head tilt. This agrees with [28]. The significant interaction between position and modality suggests that the orientation of the head and body play different roles in the two tasks, although this must be interpreted carefully as SHV errors in the body tilt condition were the opposite of previously reported errors (errors tended towards the direction of tilt, not away from it). The differential effects of position on SVV and SHV suggest these measures may rely on different information sources for their internal gravity estimate. That is, the SHV seems to be less dependent than SVV on changes to information coming from perceived head position. This difference between SVV and SHV measures indicates that the tasks may not be analogous as has been suggested [2]. It further suggests that the two tasks may serve complementary roles in measuring gravity perception, by probing different aspects of internal gravity estimates.

The model of gravity perception proposed by Clemens and colleagues [3] posits two internal estimates of gravitational vertical, one based on an estimation of the head’s position in space, and the other based on body position. Importantly, these two estimates may inform each other via the “indirect pathway” (Fig 1), whereby the perceived position of the neck is used to convert one estimate into the reference frame of the other. Previous work [3,29,30] has made a strong case for SVV relying on a head-based estimate of gravity vertical, while our results suggest a role for the indirect pathway in this task as well. Our data also support the theory that SHV accesses a body-based estimate.

It has been argued that the bias driving SVV A-effects may be tied to a head-fixed reference frame [30]. If this were true, and if SVV were to rely more heavily on head position information than SHV, then introducing noise to the head-in-space estimate should have differential effects on the two measures, exacerbating the effects of the head-in-space prior (see Fig 1), while leaving SHV largely unaffected. To test this, we repeated experiment 2 while applying vestibular noise using disruptive galvanic vestibular stimulation (dGVS).

## Experiment 3: The Effect of Vestibular Noise on SVV and SHV

The otolith organs are the part of the vestibular system that senses linear acceleration, including gravitational acceleration acting on the body. They are a primary source of information about the direction of gravity, although not the only source. This sense can be disrupted by using disruptive galvanic vestibular stimulation (dGVS), in which an unpredictable electrical stimulation created by using a sum-of-sines stimulation pattern [31,32] is applied to the surface of the skin behind the ears [33] (for an image of this apparatus see the left-hand photo in Fig 3 of [34]). Importantly, the alternating sum-of-sines signal promotes a sense of instability without consistent illusory motion (such as illusory swaying or leaning, elicited by a sine wave or unilateral stimulation, respectively), thus effectively serving as vestibular “noise” [32]. If SVV relies on head-based information about gravitational vertical combined with a head-upright prior (as has been suggested by [16], and is featured in Clemens et al.’s model, shown in Fig 1) then we would expect the SVV to show a stronger bias towards that prior when the vestibular signal is compromised. Conversely, if SHV were driven by characteristics of the body and limbs then vestibular noise may not alter SHV estimates. In this study we repeated experiment 2 with the addition of vestibular noise via dGVS.

### Methods

#### Participants

15 participants (aged 18–36, 14 female) took part in this experiment. Two of the participants had completed the previous experiment, while the rest were naïve. Two of the participants identified themselves as left-handed and one identified as ambidextrous.

#### Galvanic Vestibular Stimulation

Vestibular noise was applied using a galvanic vestibular stimulator (Good Vibrations Engineering Ltd., Nobleton, Ontario, Canada) connected via the serial port to a computer through which it was controlled by a MATLAB program. Two electrodes (1.25” dia, 9000 series, Empi Recovery Sciences, St. Paul, Minnesota, USA) were placed on the mastoid process behind each ear and secured with tape. The stimulator was programmed to send an alternating sum-of-sines voltage with dominant frequencies at 0.16, 0.33, 0.43 and 0.61 Hz between electrodes (maximum current of 5 ma, sampling rate 25 ms). Stimulation was bipolar such that one electrode emitted the inverse voltage of the other electrode. The specific parameters and pattern of stimulation were taken from [31] and [32] and chosen to be maximally disruptive.

Prior to testing in the dGVS condition, the dGVS was turned on briefly (for approximately 10 s) to familiarize participants with the sensation and ensure the electrodes were well attached. During this initial phase one female participant became very disoriented and could not stand unsupported. This individual was removed from the experiment immediately and did not complete any testing.

#### Procedure

The procedure was as described in experiment 2. The dGVS current began immediately when the test probe’s orientation had been reached. After a 1.5 s lag, a 400 Hz beep indicated the trial had started. Participants were instructed to judge whether the rod was tilted to the right or left of gravitational vertical (procedure described in detail above). The dGVS current was stopped when a response was made, and a 2 s interval preceded the next trial.

### Results

Results of this experiment are shown in Fig 2C. Data from this experiment were compared to those from experiment 2 (here serving as a control condition). A mixed model 2x2x2 ANOVA found a main effect of modality (SVV or SHV), *F*(1, 26) = 12.75, *p* = 0.001, of position (head or body tilt), *F*(1, 26) = 12.83, *p* = 0.001, and a main effect of dGVS, *F*(1,26) = 9.69, *p* = 0.004. There was a significant interaction between modality x position, *F*(1,26) = 21.21, *p*<0.001, and between modality x dGVS, *F*(1,26) = 7.08, *p* = 0.013. Of all of the tested conditions, only the SHV + head tilt produced an estimate of gravity that was significantly biased away from veridical, *t* (14) = 5.04, *p*<0.001. Correlation analyses between gravity estimates of SVV and SHV under dGVS were not significant for the head tilt position or for the body tilt position.

### Discussion

Experiment 2 showed that both head and body tilt induced SVV errors, while only body tilt evoked SHV errors. Here we disrupted the vestibular signal using a bipolar alternating sum-of-sines waveform to generate a sense of instability or “noise” [32] in the head-based gravity estimate. Clemens et al.’s model of gravity perception [3] (see Fig 1) predicts that this would primarily impair SVV estimates, in line with the assumption that SVV accesses the head-based estimate while we argue SHV accesses the body-based estimate. Surprisingly, dGVS eliminated SVV biases in both the head tilt and body tilt condition altogether. These results do not support the model, or the existence of a head-upright prior [16] that would be expected to dominate SVV and create a bias towards the head’s position. It is possible that the dGVS led participants to adopt a different strategy for judging head orientation, one that did not invoke a head-upright prior. A few participants reported seeing the visual probe sway erratically from side to side while experiencing dGVS, which provides anecdotal evidence to suggest dGVS may have triggered torsional movements of the eye [35,36]. It is possible that the brain adopts a different strategy for judging the verticality of a probe in motion compared to a static probe; this is plausible given that motion information and static object properties such as luminance and colour are processed in different pathways in the brain [37,38], and may be incorporated differently into verticality judgments. However, to our knowledge no study has systematically compared SVV judgments of moving versus static visual probes. Further evidence is needed to support this explanation.

Vestibular disruption evoked a strong SHV E-effect (away from tilt) when only the head was tilted, and eliminated the A-effect (towards tilt) seen in experiment 2 during body tilt. These results also do not support the reliance of SHV on a body-based estimate of gravity. Rather, our results suggest that the vestibular signal may be important for SHV after all, despite the lack of influence of head tilt on the SHV when the vestibular signal is functioning normally (experiment 2). Does this dGVS-induced bias in SHV during body tilt reflect a true shift in the corresponding underlying gravity estimate? Or might it reflect instead a shift in the perceived position of the hands? Studies have shown that vestibular stimulation shifts the location of participants’ drawings [39], induces reaching errors [40,41], leads to mislocalization of touches on the hands [42] and creates elongation and widening of the perception of the hand compared to controls [43]. The impact of dGVS on SHV might be due not to a distortion of the underlying gravity estimate but to a change in the perceived location of the hand. Thus, the error would stem from the judgment of stimulus orientation with respect to the body rather than to an error in the perceived direction of gravity.

In summary, dGVS eliminated the SVV and SHV biases normally evoked by tilt (as reported in experiment 2), but evoked a strong SHV bias when the body was upright and the head was tilted. Though the underlying impact of dGVS on body perception and strategies for judging visual verticality is still unclear, it is apparent that the addition of vestibular noise has a different effect on SVV and SHV, further supporting the claim that the two measures are driven by separate underlying mechanisms. Experiment 4 investigates whether these mechanisms are separate enough that SVV and SHV can be integrated in a statistically optimal fashion.

## Experiment 4: Do SVV and SHV Combine Optimally?

When the brain integrates two estimates of the same variable (e.g., depth or size), it often does so according to the principles of optimizing the noise in the resulting estimate. It does this by weighting the two contributing cues according to the inverse of their variance [23]. Critically, cues can only be integrated optimally *if the noise in those estimates is uncorrelated* [24]. If SHV and SVV were to access different underlying estimates of gravitational vertical, we would expect minimal correlated noise between estimates, and thus would predict optimal integration of the two measures; conversely, if the two estimates were to share a common representation of gravity, we would expect integration to be less than optimal. To test this we had participants estimate the orientation of a bar that they could simultaneously see and feel, and compared their estimates to the predictions of maximum likelihood estimation (MLE) based on unimodal performance. It quickly became apparent that when the rod was a thin, clear, internally illuminated rod, it provided a highly reliable estimate of orientation and therefore would dominate any contribution of the haptic cue. We therefore reduced the reliability of the visual cue before proceeding.

### Predicting optimal bimodal performance

Optimal bimodal verticality estimates can be predicted from unimodal performance (i.e., SVV and SHV). From these, the bimodal estimates and variances can be calculated from:

$$\frac{1}{\sigma_{\text{bimodal}}^{2}}=\;\frac{1}{\sigma_{\text{SVV}}^{2}}+\;\frac{1}{\sigma_{\text{SHV}}^{2}}$$ (1)

$${\text{PSE}}_{\text{bimodal}}={\text{w}}_{\text{SVV}}{\text{PSE}}_{\text{SVV}}+{\text{w}}_{\text{SVV}}{\text{PSE}}_{\text{SVV}}$$ (2)

$${\text{w}}_{\text{SVV}}=\;\frac{\frac{1}{\sigma_{\text{SVV}}^{2}}}{\left(\frac{1}{\sigma_{\text{SVV}}^{2}}+\frac{1}{\sigma_{\text{SHV}}^{2}}\right)}$$ (3)

$${\text{w}}_{\text{SHV}}=\;1-{\text{w}}_{\text{SVV}}$$ (4)

where PSE indicates the subjective vertical, σ2 indicates the variance and w indicates the weighting of each cue.

### Methods

#### Participants

Sixteen participants (aged 21–60, 11 female) took part in this experiment. One participant self-identified as left-handed, and one self-identified as ambidextrous.

#### Platform

Participants used the same platform described in experiment 1, tilted 45° left. That is, participants completed all tasks while at a whole body roll-tilt of -45°.

#### Test rod

In order to reduce the reliability of judging the visual probe’s orientation, a plexiglass screen covered in a layer of vehicle window tinting with 5% visible light transmission (Gila Basic Super Limo Black 5% Window Tint, http://www.gilafilms.com/en/Basic-Window-Tints.aspx) was set 30 cm in front of the test rod to blur it. The edges of the screen were not visible in the dark room. The rod was also masked with opaque tape so that only the middle 5 cm was visible. In all conditions the apparatus was arranged, as in the previous experiments, so that participants were able to reach out and explore the rod by touch.

#### Procedure

Participants completed three test blocks: (1) judging orientation by touch with the eyes closed (SHV), (2) judging orientation by sight with the blurred stimulus (SVV), and (3) judging orientation with touch and the blurred stimulus simultaneously (bimodal). Blocks were completed in a counterbalanced order for each participant. All blocks were completed in a dark room. Procedures for SVV and SHV were the same as in experiment 1. In the bimodal condition, participants were instructed to use both sight and touch to determine the orientation of the probe with respect to gravity. When the rod was illuminated, participants were instructed to reach out and feel its orientation as well as well as looking at it. Due to the presence of the blurring screen, participants could not see their own hands during haptic exploration of the probe in this condition.

Test rod orientations were selected by a Bayesian adaptive staircase (the psi-method, adapted for MATLAB from [44]), which estimated the point of subjective equivalence (PSE) and the variance (squared inverse slope) of participants’ underlying psychometric functions. The PSI method was chosen rather than the QUEST because it is optimized to accurately estimate the slope of the psychometric curve, i.e. within-subject precision. The testing algorithm was given an initial PSE estimate of 0° (no bias), a standard deviation of ±10°, a variance estimate of ±5 degrees^2^ with a standard deviation of ±10 degrees^2^, and a step size of 0.1°. Each condition yielded a PSE and a *σ* (standard deviation) score for each testing block. For each of the five testing blocks, the psi algorithm ran 150 trials, which took approximately 20 minutes. Participants completed the blocks over a period of several days.

### Results

#### PSEs

**Fig 3:**
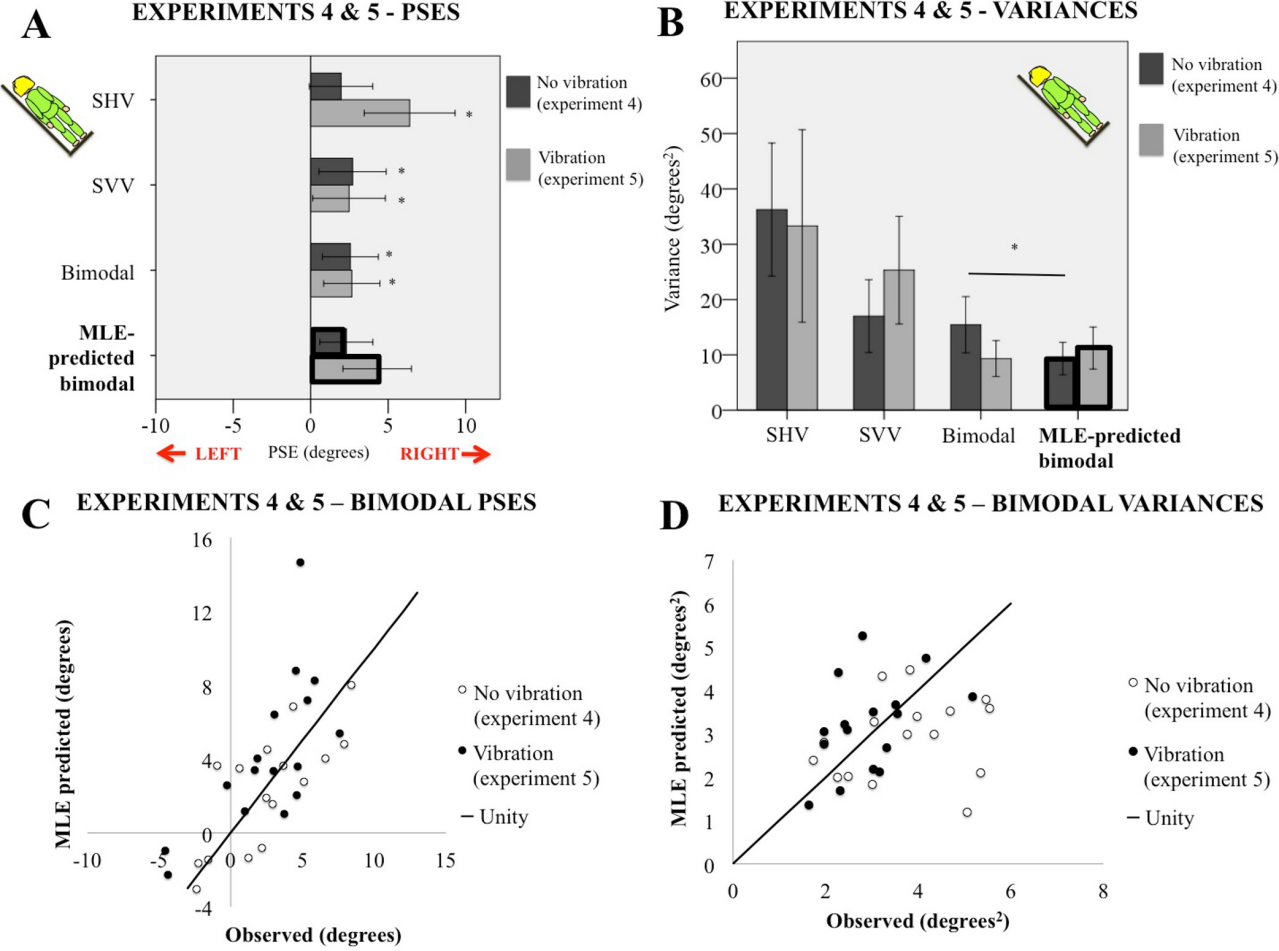
Results for experiments 4 and 5. A. Mean PSEs for SVV (with a blurry visual stimulus), SHV and bimodal measures for experiment 4 (dark grey bars), and experiment 5 (with neck vibration, light grey bars). PSEs predicted by the maximum likelihood estimate (MLE) model for each experiment are bolded. 0° corresponds to gravitational vertical. Asterisks indicate mean was significantly different from 0° at p<0.05. Error bars are 95% confidence intervals. B. Mean variance for all three modalities, with and without neck vibration. Error bars are 95% confidence intervals. MLE-predicted values are outlined in bold. C. Observed bimodal PSEs versus MLE predicted bimodal PSEs for each participant, for experiment 4 (open circles) and experiment 5 (filled circles). Solid line indicates a slope of 1. D. Observed variances of the bimodal measure versus predicted variances, for experiment 4 (open circles) and experiment 5 (filled circles). Solid line indicates a slope of 1.

The mean PSEs for SHV, SVV, and the bimodal condition are plotted with 95% confidence intervals in light grey in Fig 3A. All experiments were done with the body tilted 45° to the left. A repeated-measures ANOVA comparing PSE across the three conditions found no main effect of condition (p = 0.64). There was no correlation between SVV and SHV PSEs (*p* = 0.88). Blurring the visual stimulus resulted in a bias *away* from the body in the unimodal SVV condition, *t*(15) = 2.66, *p* = 0.018, in contrast to the SVV results from experiments 1 and 2. The bimodal PSE was also significantly biased opposite to body tilt, *t*(15) = 3.02, *p* = 0.009. SHV PSEs did not statistically differ from 0°.

The predicted value for the bimodal PSE is shown in bold (light grey) in Fig 3A, and observed and predicted scores are plotted against each other in light grey in Fig 3C. A paired t-test between the experimentally measured bimodal PSE scores and the MLE predicted scores (as calculated by Eqs 1–4 above) found no difference. Observed and predicted scores were significantly correlated, *r*(14) = 0.76, *p* = 0.001. (Note that the correct testing procedure for comparing model estimates to raw scores is an equivalence test, however, here we used standard paired Student’s t-tests instead, to avoid setting an arbitrary boundary for ‘equivalence.’ Cribbie et al. have shown that for small sample sizes, a t-test is just as or more effective at detecting statistical equivalence compared to equivalence tests [45].)

#### Variances

Mean variances (precision, σ^2^) for each condition are plotted with 95% confidence intervals in Fig 3B in light grey. A repeated-measures ANOVA found a main effect of modality on precision, *F*(2,30) = 8.59, *p*<0.001. Precision estimates for SVV and SHV were not correlated across participants. T-tests (corrected for False Discovery Rate) found that bimodal variances were significantly better than unimodal haptic variances, *t*(15) = 3.45, *p* = 0.004, but were not different from unimodal visual-alone variances. Observed versus MLE predicted bimodal variance scores are plotted in Fig 3D. Measured bimodal variance scores were significantly greater than those predicted by the model (Eq 1), *t*(15) = 2.42, *p* = 0.029, thus integration of haptic and visual estimates of gravitational vertical was not optimal. Furthermore, observed and predicted variances were not significantly correlated.

### Discussion

Measuring perceived vertical bimodally resulted in a PSE that was consistent with optimal integration as predicted by MLE, but did not show the optimal reduction of variance. The PSE findings are difficult to interpret. On average, both SHV and SVV unimodal PSEs are in the same direction and of a similar magnitude. Therefore, it would require a great deal of statistical power to distinguish an optimal averaging of such PSEs from a non-optimal response (e.g., capture, non-optimal weighting of averages). Thus our PSE results must be considered carefully, and we suggest that variance scores may be a better indicator of potential optimal integration.

In this case, the bimodal scores did not reflect optimal integration. Optimal integration requires that SVV and SHV are uncorrelated (i.e., have access to independent measures of gravitational vertical). A lack of integration between the SVV and SHV is compatible with either the two probes accessing a common gravity estimate (and hence correlated noise), or an otherwise failure of the brain to optimally combine the two cues.

#### One or two representations of gravity?

Bimodal estimates of gravity using a blurry visual stimulus showed significantly worse precision (higher variances) than predicted by MLE. This is consistent with a previous research finding that a bimodal (visual/haptic) estimate of a rod’s orientation with respect to the head produced significantly greater variance than predicted by MLE [46]. Braem et al. looked at integration of a bimodal visual/haptic vertical while participants were seated upright and reported that bimodal variance was consistent with MLE predictions [47]. However, in their experiments the bimodal variance did not differ from unimodal SVV variance, making it impossible to distinguish optimal integration from a total reliance on visual information. Our results are compatible with optimal integration being made impossible due to a common source of noise between the visual (SVV) and haptic (SHV) estimates of gravitational vertical. This is consistent with the possibility of a single shared estimate of the orientation of gravitational vertical. However, it is also possible that the two measures access different estimates of verticality and the shared noise arises from other common task elements, (for example a prior which influences both estimates in the same way, such as the idiotropic or body midline prior proposed by [15]). If such a prior did indeed have a common influence on these separate measures we would anticipate a correlation between SVV and SHV errors. Such a correlation was not found in the previous experiments (see above).

Another possibility is that the gravity estimates come primarily from separate sources, but that information can be shared between the two pathways. In their model, Clemens et al. [3] proposed an “indirect” pathway that converts body-based cues into head-based coordinates, and vice versa. This pathway is mediated by knowledge of the position of the neck (see Fig 1). By allowing for shared information between the head and the body, the indirect pathway could result in both head-based and body-based estimates of gravity contributing to SVV and SHV, thus constituting a source of shared noise. In experiment 5 we temporarily separated the functional connection between the head and body by adding noise to the neck proprioceptors making it possible for optimal integration to occur.

#### Sharp vs. blurry: a reversal of SVV errors?

Before we continue with our main theme, we feel it is worthwhile to take a little detour to discuss the surprising differences we noted in the SVV depending on whether a sharp or blurred line was used as the stimulus. The sharply visible rod used in experiments 1–3 generally evoked an SVV biased towards the body during a tilt (Fig 2), whereas a blurred, degraded stimulus evoked a bias away from the tilt (Fig 3). Pilot data collected using a sharply visible SVV task with the psi-function found similar SVV results to experiment 1 (-2.6° ± 0.8°), suggesting this bias was driven by the change of stimulus, not the change in testing paradigm. This observation raises further speculation about the prior that has been suggested to shift estimates towards the body midline [16]. Such a prior would induce consistent bias in estimates independent of the visual stimulus. Why might specific visual stimulus characteristics promote different errors in visual verticality perception? Wade [48] and Luyat et al. [49] tested SVV using different test line lengths and both studies found that longer lines elicited biases towards the body while shorter lines evoked biases in the opposite direction. This is consistent with our findings since the blurred line was also considerably shorter than the sharply visible line used in experiments 1–3 (37° sharp, 7° blur). Luyat and colleagues [49] suggest that this reversal of error may be due to additional eye movements evoked by a larger stimulus (one expanding into the periphery) creating additional cortical activation. A comprehensive model of gravity perception must take such movements into account, and clarify what stimulus characteristics invoke a head-upright prior when judging verticality. Further study on this topic is needed.

## Experiment 5: Do SVV and SHV Combine Optimally when the Head and Body Cannot Share Gravity Information?

**Fig 4:**
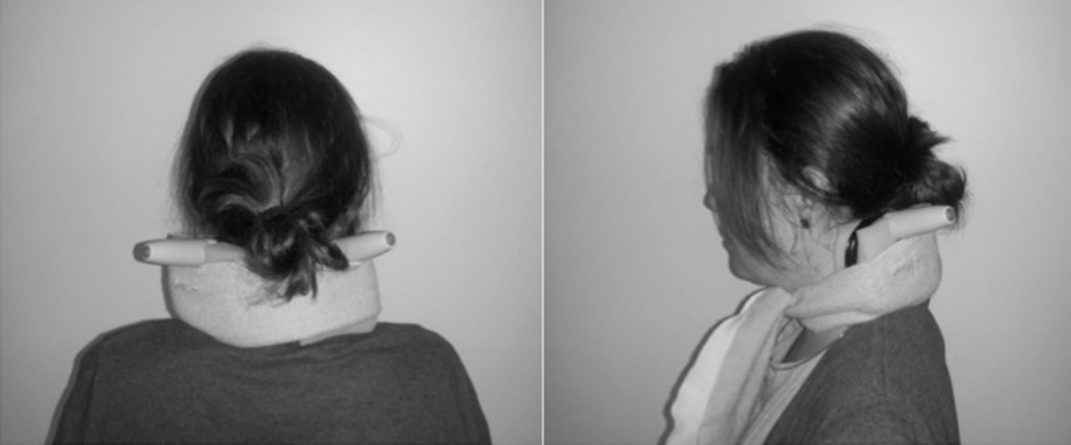
An image of the vibration apparatus. Bilateral vibration of the dorsal neck muscles was applied using two handheld vibrators embedded in foam blocks that were secured with a tensor bandage. The individual photographed has given written informed consent (as outlined in PLOS consent form) to publish this image.

In experiment 4 we aimed to test the models of Schuler et al. [2] and Clemens et al. [3] by identifying whether there was shared noise in SVV and SHV. Our failure to optimally integrate SVV and SHV could be due to shared noise between the two measures, possibly due to a shared underlying gravity estimate in the two tasks. However, Clemens et al.’s [3] two-estimate model states that head-based and body-based sensory information regarding gravity can in fact be shared via an “indirect pathway” mediated by neck proprioception (i.e., the head-on-body estimate, see Fig 1), which could also produce shared noise in the bimodal estimate. If this is the case then degrading this proprioceptive neck information should lead to a degraded indirect pathway, thus effectively ‘decoupling’ the two estimates. Here we tested SHV, blurry SVV, and the bimodal combination of the two measures while mild vibration was applied to the dorsal muscles of the upper neck (see Fig 4). This group of muscles has been implicated in head roll [50] and vibration of one side of this area affects SVV as though the head were rolled toward the side opposite the vibration, while vibration of other nearby areas did not [51]. We posited that vibration of both sets of these muscles would introduce noise into the afferent signal associated with perception of head-on-body roll and that any tendency to evoke an actual head movement percept would be cancelled out because of the bilateral application. This is, as far as we are aware, a novel application of neck muscle vibration. During the experiment, participants reported that the vibration was somewhat uncomfortable but not intolerable. If the bimodal condition were to show suboptimal integration in the control, no-vibration conditions but optimal integration in the neck vibration condition, this would be compatible with the idea that Clemens et al.’s indirect pathway was the source of shared noise in the control data, thus providing evidence for a two-estimate model of gravity perception. If suboptimal integration were to persist in the presence of vibration, it would suggest that the common source of noise preventing integration might be due to a shared underlying estimate of gravitational vertical.

### Methods

#### Participants

Sixteen participants (9 female, aged 21–60) completed this experiment. One participant self-identified as left handed, and an additional participant identified as ambidextrous.

#### Neck Vibration

Vibration was applied to both sides of the upper dorsal neck via a pair of commercial handheld battery-operated vibrators (http://www.trojanvibrations.com/product/-vibrators-pulse-vibrator.do), which were embedded in foam blocks and held in place with a tensor bandage tied securely around the participant’s neck (see Fig 4). The vibrators both had a circular surface of contact approximately 2.5 cm in diameter and a vibration frequency of 30 Hz (measured using a strobe light). They were placed on the upper dorsal neck muscles, approximately 2.5 cm below the base of the skull and 2.5 cm out to either side from the spine. In order to ensure consistent vibration intensity over time, the batteries were changed after every 2^nd^ participant. The vibrators were used at their lowest continuous vibration setting and were left on for the duration of each experimental block. The vibrators were turned off between experimental blocks.

#### Procedure

Procedure was identical to experiment 4. Participants completed all blocks over a period of several days.

### Results

#### PSEs

The mean PSEs from the SHV, SVV, and bimodal conditions are plotted with 95% confidence intervals in Fig 3A in dark gray. A repeated measures ANOVA found a significant main effect of condition *F*(2,30) = 7.03, *p =* 0.003. There was no correlation between SVV and SHV PSEs (*p* = 0.11). T-tests comparing PSEs to veridical found all three conditions produced a significant bias opposite body tilt: *t*(15) = 4.65, *p*<0.001 (SHV); *t*(15) = 2.25, *p* = 0.040 (SVV); and *t*(15) = 3.11, *p* = 0.007 (bimodal).

The predicted values for the bimodal PSE is shown in bold (dark grey) in Fig 3A. A paired t-test found that the experimentally measured bimodal PSEs almost significantly differed from MLE predicted scores (t(15) = -2.09, p = 0.053). Observed versus predicted scores are plotted in Fig 3C; the two scores were significantly correlated, *r*(14) = 0.68, *p* = 0.004.

#### Variances

Mean variances (σ^2^) for each condition are plotted with 95% confidence intervals in Fig 3B in dark grey. A repeated-measures ANOVA found a main effect of modality on precision, *F*(2,30) = 5.78, *p* = 0.008. Precision for SVV and SHV was not correlated across participants (*p* = 0.85). Corrected paired t-tests found that bimodal variances were significantly more precise than both unimodal haptic and unimodal visual variances (*t*(15) = 3.37, *p* = 0.004, and *t*(15) = 3.54, *p* = 0.003, respectively. A paired t-test found no difference between MLE-predicted and actual bimodal variance (*p* = 0.33) thus suggesting optimal integration of haptic and visual estimates of gravitational vertical. However, while MLE predicted and observed variances in experiment 5 were more strongly related than in experiment 4, they were not significantly correlated. The fan-shaped spread of the data (Fig 3D) suggests this insignificant finding might be due to insufficient statistical power.

### Discussion

In the presence of neck vibration that disrupted proprioceptive information concerning the head’s position on the body, the bimodal probe showed increased precision compared to that of each of the unimodal levels and was consistent with MLE predictions. This suggests that, in the absence of clear cues to neck position, SVV and SHV may indeed be optimally integrated. Given this finding, it seems that the reason why SVV and SHV did not integrate optimally in experiment 4 was because the two estimates had a correlated source of noise, mediated through proprioception of the neck.

In experiment 4, SHV and SVV unimodal PSEs were so close together in direction and magnitude that our experiment likely did not have the statistical power to detect a difference between optimal and non-optimal integration of the two probes. In this experiment, neck vibration generated a profound E-effect (bias away from tilt) in the SHV (Fig 3A) but did not change SVV responses. We saw a similar profound SHV E-effect in experiment 3 when the body was tilted and the vestibular signal was disrupted. We suggest that this E-effect during neck vibration may be similarly due to the absence of vestibular information refining perception of the hands, this time because head-based gravity information was dissociated from the SHV task via neck vibration.

The larger difference between the unimodal PSEs made the comparison between MLE predictions of bimodal PSEs and actual scores more statistically powerful. We found an almost-significant difference between the model and observed scores (*p* = 0.053). The reason for this difference may be related to visual feedback in the bimodal task regarding hand position. That is, it was possible to see the hand as a dark, blurry blob if it fell in front of the illuminated line while making the bimodal judgment. While this feedback likely did not change the perceived orientation of gravity as sensed by the head or the trunk, it may have refined the sense of the hand’s position in space, thus informing the haptic estimate of the orientation of the *probe*. This refined orientation would then be compared to the sensed direction of gravity, yielding a different PSE than one measured totally in the dark. Our results showing optimal integration of bimodal variance suggests that this effect primarily shifted the haptic perception of the probe’s position, and did not constitute a shared source of noise across SVV and SHV components of the bimodal task.

Our results of bimodal variance support the model proposed by Clemens and colleagues [3], in which two independent estimates of gravitational vertical share information via information about neck position. In the same article the authors provide evidence that SVV preferentially accesses the estimate based on head position; results from our second and fifth experiments suggest SHV is most likely to accesses the estimate based on body position.

## General Discussion

### A single estimate of gravity?

Could our results be compatible with a single, underlying estimate of gravity? Given that decoupling head- and body-based reference frames led to a highly distorted SHV, perhaps it is the case that the SHV does rely on the same, head-based representation of gravity as the SVV, but is simply unable to access this estimate in our experimental task. However, we would expect that such an inability to access a gravitational estimate would result in worse precision in the haptic SHV task. As Fig 3B shows, no such increase in variability occurred when comparing the SHV results of experiment 4 (no vibration) with experiment 5 (vibration) (see Fig 3B). Thus our data are not consistent with SVV and SHV accessing a single internal estimate of gravitational vertical. It seems more likely that there is a second, body-based representation of gravity being accessed by SHV that is simply less accurate, or systematically biased, when parts of the body (i.e., the trunk) are tilted.

### Why two?

Why might two separate but interactive representations of gravity co-exist? It may be due to a natural division in the sensory information transduced by the head (vestibular system) and by the body (somatosensation, somatic graviception; [52]). Indeed, Bronstein et al. [53] report that vestibular nuclear lesions lead to a distortion of SVV, but not SHV. Some researchers have speculated there may be a corresponding division in cortical pathways: thalamo-parietal projections appear to be involved in somatic graviception [54], while insular regions play more of a role in vestibular graviception [8]. The perception of gravity in body-centric and head-centric coordinates thus appear to follow distinct neural pathways, although our findings suggest that when a reliable vestibular cue is present, the two estimates share information from the relevant sensory inputs.

### A two-estimate model of gravity perception

In conclusion, our data support the existence of two internal estimates of gravitational vertical, one head-based and body-based, similar to the model proposed by Clemens et al. [3] (see Fig 1). We provided further evidence that SVV may preferentially access the head-based estimate, and make the novel assertion that SHV preferentially accesses the body-based estimate (although much of its accuracy may come from the “indirect” pathway and head-based vestibular input). We also showed bilateral vibration of the neck can degrade the pathway sharing information between these two estimates, providing a potential method for examining the two in relative isolation.

Our data are not consistent with the priors laid out in the original model, priors that state that head and body are usually upright, and thus shift the perceived orientation of gravity in the same direction as the tilt. We would expect these priors to grow stronger in the presence of sensory noise, and they do not (experiment 3); they also are not consistent across different visual stimuli (compare experiments 1 and 4). We suggest that this may be due to different strategies being adopted when judging the verticality of moving, or blurry, visual probes, as compared to static, sharply visible ones. The original priors outlined in the model may need to be refined or re-characterized such that they reflect these possible differences in visual judgment strategies.

The SVV has been used as a clinical diagnostic tool for measuring vestibular function [55,56]. Our findings highlight a distinct but complementary role for SHV in testing somatograviceptive function, and provide a methodology (bilateral neck vibration) that might be used to dissociate between head-based and body-based gravity estimates in patient populations.

## References

[1] Rock I, Heimer W. The effect of retinal and phenomenal orientation on the perception of form. Am J Psychol. JSTOR; 1957; 493–511. 13487820

[2] Schuler JR, Bockisch CJ, Straumann D, Tarnutzer AA. Precision and accuracy of the subjective haptic vertical in the roll plane. BMC Neurosci. BioMed Central Ltd; 2010;11: 83 10.1186/1471-2202-11-83 20630097 PMC2912915

[3] Clemens I a H, De Vrijer M, Selen LPJ, Van Gisbergen J a M, Medendorp WP. Multisensory processing in spatial orientation: an inverse probabilistic approach. J Neurosci. 2011;31: 5365–5377. 10.1523/JNEUROSCI.6472-10.2011 21471371 PMC6622694

[4] Aubert H. Eine scheinbare bedeutende Drehung von Objecten bei Neigung des Kopfes nach rechts oder links. Arch für Pathol Anat und Physiol und für Klin Med. 1861;20: 381–393. 10.1007/BF02355256

[5] Friedmann G. The judgement of the visual vertical and horizontal with peripheral and central vestibular lesions. Brain. 1970;93: 313–328. 10.1093/brain/93.2.313 5310320

[6] Howard IP. Human visual orientation John Wiley & Sons; 1982.

[7] Ceyte H, Cian C, Trousselard M, Barraud PA. Influence of perceived egocentric coordinates on the subjective visual vertical. Neurosci Lett. 2009;462: 85–88. 10.1016/j.neulet.2009.06.048 19545600

[8] Barra J, Marquer A, Joassin R, Reymond C, Metge L, Chauvineau V, et al Humans use internal models to construct and update a sense of verticality. Brain. Oxford Univ Press; 2010;133: 3552–3563. 10.1093/brain/awq311 21097492

[9] Van Beuzekom AD, Van Gisbergen JAM. Properties of the internal representation of gravity inferred from spatial-direction and body-tilt estimates. J Neurophysiol. Am Physiological Soc; 2000;84: 11–27. 10899179 10.1152/jn.2000.84.1.11/F

[10] De Vrijer M, Medendorp WP, Van Gisbergen JAM. Accuracy-precision trade-off in visual orientation constancy. J Vis. 2009;9: 9.1–15. 10.1167/9.2.9 19271919

[11] Tarnutzer AA, Bockisch C, Straumann D, Olasagasti I. Gravity dependence of subjective visual vertical variability. J Neurophysiol. 2009;102: 1657–1671. 10.1152/jn.00007.2008 19571203

[12] Muller GE t. Uber das Aubertsche Phanomen. Z Sinnesphysiol. 1916;49: 109–244.

[13] Wade NJ. Visual orientation during and after lateral head, body, and trunk tilt. Percept Psychophys. Springer; 1968;3: 215–219.

[14] Betts GA, Curthoys IS. Visually perceived vertical and visually perceived horizontal are not orthogonal. Vision Res. Elsevier; 1998;38: 1989–1999. 9797945 10.1016/s0042-6989(97)00401-x

[15] Mittelstaedt H. A new solution to the problem of the subjective vertical. Naturwissenschaften. 1983;70: 272–281. 10.1007/BF00404833 6877388

[16] MacNeilage PR, Banks MS, Berger DR, Bülthoff HH. A Bayesian model of the disambiguation of gravitoinertial force by visual cues. Exp Brain Res. 2007;179: 263–290. 10.1007/s00221-006-0792-0 17136526

[17] de Graaf B, Bekkering H, Erasmus C, Bles W. Influence of visual, vestibular, cervical and somatosensory tilt information on ocular rotation and perception of the horizontal. J Vestib Res Equilib Orientat. IOS Press; 1992;2: 15–30. 1342382

[18] Wade SW, Curthoys IS. The effect of ocular torsional position on perception of the roll-tilt of visual stimuli. Vision Res. Elsevier; 1997;37: 1071–1078. 9196725 10.1016/s0042-6989(96)00252-0

[19] Mast FW. Does the world rock when the eyes roll? Allocentric orientation representation, ocular counterroll, and the subjective visual vertical. Swiss J Psychol Zeitschrift f{ü}r Psychol Suisse Psychol. Verlag Hans Huber; 2000;59: 89.

[20] Bauermeister M, Werner H, Wapner S. The effect of body tilt on tactual-kinesthetic perception of verticality. Am J Psychol. JSTOR; 1964; 451–456. 14198668

[21] Bortolami SB, Pierobon A, DiZio P, Lackner JR. Localization of the subjective vertical during roll, pitch, and recumbent yaw body tilt. Exp Brain Res. 2006;173: 364–373. 10.1007/s00221-006-0385-y 16628401

[22] Tarnutzer AA, Schuler JR, Bockisch CJ, Straumann D. Hysteresis of haptic vertical and straight ahead in healthy human subjects. BMC Neuroscience. 2012 p. 114 10.1186/1471-2202-13-114 22998034 PMC3505461

[23] Ernst MO, Banks MS. Humans integrate visual and haptic information in a statistically optimal fashion. Nature. 2002;415: 429–433. 10.1038/415429a 11807554

[24] Ernst MO. Optimal multisensory integration: Assumptions and limits In: Stein BE, editor. The new handbook of multisensory processes. Cambridge: MIT Press; 2012 pp. 527–544.

[25] Baccini M, Paci M, Del Colletto M, Ravenni M, Baldassi S. The assessment of subjective visual vertical: comparison of two psychophysical paradigms and age-related performance. Atten Percept Psychophys. 2014;76: 112–22. 10.3758/s13414-013-0551-9 24092357

[26] Watson AB, Pelli DG. QUEST: a Bayesian adaptive psychometric method. Percept Psychophys. 1983;33: 113–120. 10.3758/BF03202828 6844102

[27] Tesio L, Longo S, Rota V. The subjective visual vertical: validation of a simple test. Int J Rehabil Res. LWW; 2011;34: 307–315. 10.1097/MRR.0b013e32834c45bc 21959121

[28] Tarnutzer AA, Bockisch CJ, Olasagasti I, Straumann D. Egocentric and allocentric alignment tasks are affected by otolith input. J Neurophysiol. Am Physiological Soc; 2012;107: 3095–3106. 10.1152/jn.00724.2010 22442575

[29] Guerraz M, Poquin D, Luyat M, Ohlmann T. Head orientation involvement in assessment of the subjective vertical during whole body tilt. Percept Mot Skills. Ammons Scientific; 1998;87: 643–648. 9842617 10.2466/pms.1998.87.2.643

[30] Tarnutzer AA, Bockisch CJ, Straumann D. Roll-dependent modulation of the subjective visual vertical: contributions of head- and trunk-based signals. J Neurophysiol. 2010;103: 934–941. 10.1152/jn.00407.2009 20018837

[31] Moore ST, MacDougall HG, Peters BT, Bloomberg JJ, Curthoys IS, Cohen HS. Modeling locomotor dysfunction following spaceflight with galvanic vestibular stimulation. Exp brain Res. Springer; 2006;174: 647–659. 16763834 10.1007/s00221-006-0528-1

[32] MacDougall HG, Moore ST, Curthoys IS, Black FO. Modeling postural instability with Galvanic vestibular stimulation. Exp brain Res. Springer; 2006;172: 208–220. 16432695 10.1007/s00221-005-0329-y

[33] Fitzpatrick RC, Day BL. Probing the human vestibular system with galvanic stimulation. J Appl Physiol. Am Physiological Soc; 2004;96: 2301–2316. 15133017 10.1152/japplphysiol.00008.2004

[34] Trainor LJ, Gao X, Lei J, Lehtovaara K, Harris LR. The primal role of the vestibular system in determining musical rhythm. Cortex. 2009;45: 35–43. 10.1016/j.cortex.2007.10.014 19054504

[35] Schneider E, Glasauer S, Dieterich M. Comparison of human ocular torsion patterns during natural and galvanic vestibular stimulation. J Neurophysiol. 2002;87: 2064–2073. 10.1152/jn.00558.2001 11929924

[36] Séverac Cauquil A, Faldon M, Popov K, Day BL, Bronstein AM. Short-latency eye movements evoked by near-threshold galvanic vestibular stimulation. Exp Brain Res. 2003;148: 414–418. 10.1007/s00221-002-1326-z 12541151

[37] Maunsell JH, Newsome WT. Visual processing in monkey extrastriate cortex. Annu Rev Neurosci. 1987;10: 363–401. 10.1146/annurev.neuro.10.1.363 3105414

[38] Vaina LM. Functional segregation of color and motion processing in the human visual cortex: clinical evidence. Cereb Cortex. 1994;4: 555–572. 7833656 10.1093/cercor/4.5.555

[39] Guerraz M, Blouin J, Vercher J-L. From head orientation to hand control: evidence of both neck and vestibular involvement in hand drawing. Exp Brain Res. 2003;150: 40–49. 10.1007/s00221-003-1411-y 12698215

[40] Bresciani JP, Blouin J, Popov K, Bourdin C, Sarlegna F, Vercher JL, et al Galvanic vestibular stimulation in humans produces online arm movement deviations when reaching towards memorized visual targets. Neurosci Lett. 2002;318: 34–38. 10.1016/S0304-3940(01)02462-4 11786219

[41] Mars F, Archambault PS, Feldman AG. Vestibular contribution to combined arm and trunk motion. Exp Brain Res. 2003;150: 515–519. 10.1007/s00221-003-1485-6 12695873

[42] Ferrè ER, Bottini G, Haggard P. Vestibular modulation of somatosensory perception. Eur J Neurosci. 2011;34: 1337–1344. 10.1111/j.1460-9568.2011.07859.x 21978189

[43] Lopez C, Schreyer H-M, Preuss N, Mast FW. Vestibular stimulation modifies the body schema. Neuropsychologia. Elsevier; 2012;50: 1830–1837. 10.1016/j.neuropsychologia.2012.04.008 22561888

[44] Kontsevich LL, Tyler CW. Bayesian adaptive estimation of psychometric slope and threshold. Vision Res. 1999;39: 2729–2737. 10.1016/S0042-6989(98)00285-5 10492833

[45] Cribbie RA, Gruman JA, Arpin-Cribbie CA. Recommendations for applying tests of equivalence. J Clin Psychol. Wiley Online Library; 2004;60: 1–10. 14692005 10.1002/jclp.10217

[46] Gueguen M, Vuillerme N, Isableu B. Does the integration of haptic and visual cues reduce the effect of a biased visual reference frame on the subjective head orientation? PLoS One. Public Library of Science; 2012;7: e34380 10.1371/journal.pone.0034380 22509295 PMC3324492

[47] Braem B, Honoré J, Rousseaux M, Saj a., Coello Y. Integration of visual and haptic informations in the perception of the vertical in young and old healthy adults and right brain-damaged patients. Neurophysiol Clin. Elsevier Masson SAS; 2014;44: 41–48. 10.1016/j.neucli.2013.10.137 24502904

[48] Wade NJ. The effect of stimulus line variations on visual orientation with head upright and tilted. Aust J Psychol. Wiley Online Library; 1969;21: 177–185.

[49] Luyat M, Noël M, Thery V, Gentaz E. Gender and line size factors modulate the deviations of the subjective visual vertical induced by head tilt. BMC Neurosci. BioMed Central Ltd; 2012;13: 28 10.1186/1471-2202-13-28 22420467 PMC3329413

[50] Mayoux-Benhamou MA, Revel M, Vallee C. Selective electromyography of dorsal neck muscles in humans. Exp brain Res. Springer; 1997;113: 353–360. 9063721 10.1007/BF02450333

[51] McKenna GJ, Peng GCY, Zee DS. Neck muscle vibration alters visually perceived roll in normals. J Assoc Res Otolaryngol. Springer; 2004;5: 25–31. 14569429 10.1007/s10162-003-4005-2 PMC2538369

[52] Mittelstaedt H. Somatic graviception. Biol Psychol. Elsevier; 1996;42: 53–74. 8770370 10.1016/0301-0511(95)05146-5

[53] Bronstein AM, Perennou DA, Guerraz M, Playford D, Rudge P. Dissociation of visual and haptic vertical in two patients with vestibular nuclear lesions. Neurology. AAN Enterprises; 2003;61: 1260–1262. 14610132 10.1212/01.wnl.0000086815.22816.dc

[54] Perennou D a., Mazibrada G, Chauvineau V, Greenwood R, Rothwell J, Gresty M a., et al Lateropulsion, pushing and verticality perception in hemisphere stroke: a causal relationship? Brain. 2008;131: 2401–2413. 10.1093/brain/awn170 18678565

[55] Anastasopoulos D, Haslwanter T, Bronstein A, Fetter M, Dichgans J. Dissociation between the perception of body verticality and the visual vertical in acute peripheral vestibular disorder in humans. Neurosci Lett. Elsevier; 1997;233: 151–153. 9350855 10.1016/s0304-3940(97)00639-3

[56] Vibert D, Hausler R, Safran AB. Subjective visual vertical in peripheral unilateral vestibular diseases. J Vestib Res. IOS Press; 1999;9: 145–152. 10378186

